# Response Inhibition and Error Monitoring during a Visual Go/No-Go Task in Inuit Children Exposed to Lead, Polychlorinated Biphenyls, and Methylmercury

**DOI:** 10.1289/ehp.1103828

**Published:** 2011-12-05

**Authors:** Olivier Boucher, Matthew J. Burden, Gina Muckle, Dave Saint-Amour, Pierre Ayotte, Éric Dewailly, Charles A. Nelson, Sandra W. Jacobson, Joseph L. Jacobson

**Affiliations:** 1 Centre de recherche du Centre Hospitalier Universitaire de Québec, Québec, Canada; 2 Department of Psychiatry and Behavioral Neurosciences, Wayne State University School of Medicine, Detroit, Michigan, USA; 3 Université Laval, Québec, Canada; 4 Centre de recherche, Hôpital Sainte-Justine, Montréal, Québec, Canada; 5 Département de psychologie, Université du Québec à Montréal, Montréal, Québec, Canada; 6 Children’s Hospital Boston/Harvard Medical School, Boston, Massachusetts, USA

**Keywords:** event-related potentials, error monitoring, executive function, lead, methylmercury, neurotoxicity, polychlorinated biphenyls, response inhibition

## Abstract

Background: Lead (Pb) and polychlorinated biphenyls (PCBs) are neurotoxic contaminants that have been related to impairment in response inhibition.

Objectives: In this study we examined the neurophysiological correlates of the response inhibition deficits associated with these exposures, using event-related potentials (ERPs) in a sample of school-age Inuit children from Arctic Québec exposed through their traditional diet.

Methods: In a prospective longitudinal study, we assessed 196 children (mean age, 11.3 years) on a visual go/no-go response inhibition paradigm. Pb, PCB, and mercury (Hg) concentrations were analyzed in cord and current blood samples. Hierarchical multiple regression analyses were conducted to examine the associations of contaminant levels to go/no-go performance (mean reaction time, percent correct go, percent correct no-go) and five ERPs [N2, P3, error-related negativity, error positivity (Pe), and correct response positivity (Pc)] after control for confounding variables.

Results: Current blood Pb concentrations were associated with higher rates of false alarms and with decreased P3 amplitudes to go and no-go trials. Current plasma PCB-153 concentrations were associated with slower reaction times and with reduced amplitudes of the Pe and Pc response-related potentials. Hg concentrations were not related to any outcome on this task but showed significant interactions with other contaminants on certain outcomes.

Conclusions: These results suggest that Pb exposure during childhood impairs the child’s ability to allocate the cognitive resources needed to correctly inhibit a prepotent response, resulting in increased impulsivity. By contrast, postnatal PCB exposure appears to affect processes associated with error monitoring, an aspect of behavioral regulation required to adequately adapt to the changing demands of the environment, which results in reduced task efficiency.

Lead (Pb) and polychlorinated biphenyls (PCBs) are widespread environmental contaminants known for their developmental neurotoxicity. Despite differences in their chemical structures and properties, early exposure to these pollutants appears to produce similar effects on neurobehavior, including impulsivity, hyperactivity, and impairment in executive function (Boucher et al. 2009a; Brockel and Cory-Slechta 1998; Nicolescu et al. 2010; Rice 1999). These effects have been hypothesized to reflect dysfunction of the prefrontal cortex (Levin et al. 1992; Meng et al. 2005) and alterations in dopamine function (Seegal et al. 1997; White et al. 2007).

The term “executive function” refers to a set of cognitive processes involved in goal-directed behaviors, which are often elicited in new and/or complex situations (Norman and Shallice 1986). One key component of executive function is the ability to inhibit dominant or prepotent responses when necessary (Miyake et al. 2000), and impairment in this ability can result in impulsivity. Studies investigating the relation of Pb and PCB exposure to impulsivity and executive function have relied exclusively on neuropsychological and observational assessments. Because of their high temporal resolution, event-related potentials (ERPs) make it possible to evaluate children’s performance in specific response components arrayed across time. ERPs recorded during a task requiring response inhibition could help in understanding the neurobehavioral effects associated with Pb and PCB exposures in children.

The Inuit from Nunavik (Arctic Québec, Canada) are among the most heavily exposed populations on earth to PCBs and methylmercury (MeHg) because of the long-range transport of these chemicals via atmospheric and ocean currents and their bioaccumulation in fish and sea mammals that are staples of the traditional Inuit diet (Muckle et al. 2001). Pb exposure is also a concern because of the use of Pb cartridges for game bird hunting despite governmental regulations designed to discourage their use (Lévesque et al. 2003). This study was designed to examine the relation of exposure to these environmental contaminants to response inhibition and executive control of behavior using ERPs recorded during a visual go/no-go task in a sample of school-age Inuit children participating to a birth-cohort study. Go/no-go tasks typically require a participant to press a button in response to a given set of stimuli (“go” condition) and to inhibit that action (not to press the same button) in response to a different set of stimuli (“no-go” condition).

The go/no-go task employed in this study was developed for functional magnetic resonance imaging by Casey et al. (1997), who reported increased prefrontal cortex activity during response inhibition. The task was adapted for ERPs by Davis et al. (2003), who examined two late-latency components in relation to response inhibition, the N2 and the P3. The N2 is maximal > 300 msec poststimulus over frontal electrode locations and has been attributed to conflict detection or to the decision to withhold a motor response (Falkenstein 2006). The P3, maximal approximately 500 msec poststimulus at centroparietal electrodes, shows enhanced activity for no-go stimuli and is thought to reflect the amount of resources needed for task processing and/or efficiently inhibiting a motor response (Davis et al. 2003).

Error monitoring is another aspect of the executive control that can be assessed using ERPs. ERPs recorded after erroneous responses show two successive components, “error-related negativity” (ERN) and “error positivity” (Pe). The ERN is maximal over frontocentral electrodes approximately 80 msec after an incorrect response and is thought to reflect the dynamics of response selection and conflict (Hughes and Yeung 2011). The Pe peaks 200–400 msec after an incorrect response and is more centrally distributed; it is thought to reflect the conscious recognition, or motivational significance, of an error (Hughes and Yeung 2011; Overbeek et al. 2005). A few reports mention a similar component elicited by correct responses: the “correct response positivity” (Pc) (Burgio-Murphy et al. 2007; Laurens et al. 2010).

We hypothesized that postnatal Pb and prenatal PCB exposures would both be associated with increased impulsivity as reflected by behavioral performance in the go/no-go task and that these effects would be accompanied by alterations in five ERP components: N2, P3, ERN, Pe, and Pc. Because this is, to our knowledge, the first study to use ERPs to assess the effects of Pb and PCBs on response inhibition, no hypothesis was made concerning which components would be affected by each contaminant. Because response inhibition was not consistently associated with MeHg exposure in previous studies with children (e.g., Debes et al. 2006; Stewart et al. 2005; cf. Stewart et al. 2006), no hypothesis was put forward concerning MeHg effects in this study.

## Materials and Methods

*Participants.* The study participants were 212 school-age Inuit children from Nunavik. The Nunavik region is located north of the 55th parallel, about 1,500 km from Montreal. These children were originally recruited in the Cord Blood Monitoring Program (CBMP; 1993–1998), which was designed to document the levels of environmental contaminants and nutrients in newborns in Arctic Québec (Dallaire et al. 2001), except for one child who was recruited for the Environmental Contaminants and Child Development Study (1996–2000; Jacobson et al. 2008; Muckle et al. 2001), which was initiated during the latter part of the CBMP. Mothers were contacted by telephone, provided with information about the study protocol, and invited to participate with their children.

Assessments were conducted between September 2005 and April 2007 in the three largest Nunavik villages. Participants who resided in other communities were transported by plane to one of the larger villages for testing. A maternal interview was conducted to document information on demographic background, smoking, alcohol and drug use during pregnancy, and other maternal characteristics. The following inclusion criteria were used: age between 9.0 and 13.0 years, birth weight ≥ 2,500 g, gestation duration ≥ 35 weeks, no known neurological or clinically significant developmental disorder, and no medication for attention problems. Of the 212 participating children, 2 with a history of epilepsy, 2 with a history of head trauma associated with loss of consciousness and/or requiring hospitalization, 1 with multiple sclerosis, and 1 with a history of meningitis were excluded after data collection. Written informed consent was obtained from a parent of each participant; oral assent was obtained from each child. The research was approved by the Laval University and Wayne State University ethics committees and was performed in accordance with ethical standards of the 1983 Helsinki Declaration.

*Go/no-go protocol.* Each participant was seated 57 cm from a 43-cm flat-panel monitor on which letters were displayed centrally within a 7 × 7-cm space. The child held a button box in his or her hand and was instructed to press the button as quickly and accurately as possible with the index finger for all individually presented letters (the “go” trials) except the target “X” (the “no-go” trials). The stimuli were presented for 500 msec, with random interstimulus intervals ranging from 1,200 to 1,400 msec. The first block consisted of 40 go trials and served to prime go responses within the second block of trials. This second block consisted of 126 go trials (70%) randomly intermixed with 54 no-go trials (30%). Correct and incorrect responses were tabulated. Mean reaction time (RT; time between stimulus onset and button press) and response accuracy (percent correct) for go and no-go trials during the second block of trials were computed. Data from the initial block of 40 go trials were not analyzed (for a more detailed description of this protocol, see Burden et al. 2011).

*Electroencephalogram recording and analyses.* The electroencephalogram (EEG) was recorded with 30 Ag-AgCl electrodes placed according to the international 10–20 system (Jasper 1958) referenced online to the vertex (Cz) electrode, with forehead ground. The electro-oculogram (EOG) was recorded from bipolar miniature electrodes placed vertically above and below the right eye. Impedance was kept < 10 kΩ. EOG and EEG gain were amplified with gains of 5,000 and 50,000, respectively. The bandpass filter was 0.1–30 Hz, and a 60-Hz notch filter was engaged. The digitization rate was 200 Hz.

ERPs were derived and analyzed using Brain Vision Analyzer software (version 2.0; Brain Products, Munich, Germany). EEG channels were re-referenced offline to linked earlobes. EOG correction (Gratton et al. 1983), artifact rejection (± 100 μV), and baseline correction (100 msec) were applied. All responses occurring 200–1,600 msec after stimulus onset were considered valid. ERPs were averaged for correct go, correct no-go, and incorrect no-go responses separately. The “stimulus-locked” components, which are measured in relation to when the stimulus first appeared on the screen, were segmented 100 msec before and 1,000 msec after stimulus onset; the “response-locked” components, which are measured in relation to when the child pressed the button, were segmented 300 msec before and 500 msec after button press. Peak amplitude (microvolts) and latency to peak (milliseconds) of the stimulus-locked N2 (250–500 msec) component were identified using automatic detection. Mean amplitude values were computed for the stimulus-locked P3 (400–700) and the response-locked ERN (incorrect no-go, 0–125 msec), Pe (incorrect no-go, 100–500 msec), and Pc (correct go, 100–300 msec) components. The electrode site at which each ERP component reached its maximal amplitude was used in analyses. Participants were retained in the analyses if their behavioral performance exceeded chance level on the task and if they had a sufficient number of acceptable trials in their ERP average (≥ 12 correct no-go trials for the stimulus-locked ERPs and ≥ 8 incorrect no-go trials for the response-locked ERPs).

*Biological samples.* Umbilical cord and child blood samples were analyzed for concentrations of Pb, PCBs, mercury (Hg), and polyunsaturated fatty acids (for a detailed description of the analytical procedures, see Boucher et al. 2011). Cord blood Pb levels were determined by graphite furnace atomic absorption with Zeeman background correction. Selected PCB congeners were measured in purified cord plasma extracts using high-resolution gas chromatography with electron capture detection, and in purified child plasma extracts by high-resolution gas chromatography/mass spectrometry. PCB congener 153, expressed on a lipid basis, was used as an indicator of total PCB exposure because it is highly correlated with other PCB congeners and is considered an adequate marker of exposure to environmental PCB mixtures in the Arctic (Ayotte et al. 2003). Total Hg concentrations were determined in cord blood using cold-vapor atomic absorption spectrometry. Total Pb and Hg concentrations in child blood samples were determined by inductively coupled plasma-mass spectrometry. The limits of detection (LODs) for cord sample analyses were 0.2 μg/dL for Pb and Hg, and 0.02 μg/L for all PCB congeners in plasma. LODs for child blood sample analyses were 0.002 μg/dL for Pb, 0.1 μg/L for Hg, and < 0.05 μg/L for most PCB congeners. A value equal to half the LOD was entered in the database whenever a substance was not detected. Omega-3 fatty acid composition of plasma phospholipids was analyzed using capillary gas-liquid chromatography with flame ionization detection. Concentrations of docosahexaenoic acid (DHA) were expressed as percentages of the total area of all fatty acid peaks from C14:0 to C24:1 (percent weight).

*Control variables.* The following control variables were included: age and sex of child; whether or not the child was adopted; whether the child was transported by plane from a small, more remote village to a larger village for the assessment; time at ERP assessment (morning vs. afternoon); maternal age at delivery; socioeconomic status (Hollingshead 1975) of the primary caregiver; maternal nonverbal reasoning abilities (Raven Progressive Matrices; Raven et al. 1992); breast-feeding duration (number of months); maternal tobacco smoking (yes/no), marijuana consumption (yes/no), and binge drinking (at least one episode of ≥ 5 standard alcohol drinks; yes/no) during pregnancy; and DHA concentrations in cord and child plasma samples. In addition, in the analysis of each contaminant, the other contaminants were also treated as control variables.

*Statistical analyses.* Normality of distribution was inspected visually for each variable and checked for skewness (normality range, –3.0 to 3.0). Log transformations were performed on cord and current blood Pb, PCB-153, and Hg concentrations, breast-feeding duration, and maternal tobacco consumption during pregnancy because these variables followed log-normal distributions. Extreme values (> 3 SDs from the mean) for normally distributed independent and confounding variables were recoded to one point greater than the highest observed nonoutlying value (Winer 1971).

The relation of each behavioral and ERP outcome to Pb, PCB-153, and Hg concentrations in cord and current blood samples was examined in a series of hierarchical multiple regression analyses. Each of the control variables related at *p* < 0.20 to the outcome measure in question was entered hierarchically into a regression analysis, in which the contaminant being examined had been entered at the first step. The control variables were then entered individually using a forward selection approach (Greenland and Rothman 1998) with order of entry determined by the strength of the correlation of the control variable to the end point in question. Control variables were retained when their entry in the model altered the standardized regression coefficient for the contaminant variable by ≥ 10%. To explore possible interaction effects, all regression analyses were rerun to add the interaction terms: first for cord Pb by cord Hg, then for cord Pb by cord PCB, and then for cord Hg by cord PCB. Similar analyses with interaction terms for the three childhood contaminant measures were also run. Because such interaction terms often lack the power to detect interactions, the regression analyses for cord Pb were also rerun separately for high and low cord Hg exposure (split at the median) and then for cord PCB split at the median; the cord Hg regressions were rerun split by cord Pb and then by cord PCB; and the cord PCB regressions were rerun and split by cord Pb and then by cord Hg. Similar stratified regressions were also run for the child contaminant measures.

## Results

*Descriptive statistics.* A total of 196 participants were included in the final analyses. Reasons for exclusion were technical problems during recording (*n* = 4), too much noise in the EEG signal to produce a reliable ERP waveform (*n* = 3), and random responding on the task (*n* = 3). Compared with the retained participants, these 10 excluded participants had higher cord PCB-153 concentrations [*t*_(199)_ = 2.57, *p* = 0.01] and tended to have higher current PCB-153 [*t*_(200)_ = 1.83, *p* = 0.07] and Pb [*t*_(201)_ = 1.84, *p* = 0.07] concentrations but did not differ on any of the other exposure or control variables (all *p*-values > 0.10). When the data from the children with too much noise in the EEG signal were included in the behavioral analyses and those with random responding were included, the results remained essentially unchanged. An additional three children were excluded from analyses involving stimulus-locked components because of an insufficient number of trials in their ERP averaged waveform, and 30 were excluded from analyses involving response-locked components for the same reason. Those 33 participants excluded from either ERP analysis had higher PCB-153 [*t*_(189)_ = 3.16; *p* = 0.002] and Hg [*t*_(191)_ = 2.10; *p* = 0.04] concentrations in cord blood and higher blood Hg concentrations at testing [*t*_(191)_ = –2.42; *p* = 0.02].

Descriptive statistics of the final study sample are summarized in Table 1. Pb levels at time of testing are similar to those reported in other epidemiological studies of low-level postnatal Pb exposure at school age (e.g., Surkan et al. 2007), although the levels measured at 11 years of age in this study are about half those seen at 5 years in a subsample of these participants (Boucher et al. 2009b). Five children had blood Pb concentrations above the 10-μg/dL threshold used by U.S. and Canadian public health agencies for evaluating risk of Pb neurotoxicity and were therefore referred to the appropriate public health authorities. Boys had higher blood Pb concentrations at time of testing than did girls (3.4 vs. 2.0 μg/dL, *p* < 0.001), which probably reflects their greater involvement in hunting-related activities. Cord PCB concentrations are about three times higher than in southern Québec (Ayotte et al. 2003) and similar to those in the Dutch PCB study (Longnecker et al. 2003). Cord Hg concentrations are about 20 times higher than in southern Québec (Muckle et al. 2001) and similar to those reported in children from the Faroe Islands (Debes et al. 2006).

**Table 1:** Descriptive statistics for the study sample (*n* = 196).

Variables	*n*	Mean ± SD	Median	Range	Percent
Child age at assessment (years)		196		11.3 ± 0.6		11.4		9.8–12.9		
Child sex (% girls)		196								55.1
Adoption status (% adopted)		196								15.8
Transportation by plane (% yes)		196								45.4
Time at ERP assessment (% morning)		196								53.1
Maternal characteristics										
Age at delivery (years)		196		23.9 ± 5.8		22.0		15–42		
Marital status (% single)		195								25.1
Education (years)		196		8.3 ± 2.6		8.5		0–16		
Hollingshead score		196		28.9 ± 12.4		28.5		8.0–66.0		
Raven score		196		35.1 ± 10.1		37.0		4–56		
Breast-feeding duration*a* (months)		190		16.0 ± 17.5		11.5		0.1–108.0		68.4
Maternal use or consumption during pregnancy										
Cigarettes (% yes)		189								82.0
Marijuana (% yes)		160								21.3
Binge drinking (≥ 5 standard drinks of alcohol per occasion; % yes)		160								31.3
Contaminants and other biological samples										
Cord blood Pb (μg/dL)		193		4.8 ± 3.4		3.7		0.8–20.9		
Current blood Pb (μg/dL)		193		2.6 ± 2.2		2.0		0.4–12.8		
Cord plasma PCB-153 (μg/kg fat)		191		117.7 ± 90.9		93.3		9.7–653.6		
Current plasma PCB-153 (μg/kg fat)		192		72.0 ± 70.7		45.7		3.5–431.4		
Cord blood Hg (μg/L)		193		21.2 ± 17.6		16.6		1.0–99.3		
Current blood Hg (μg/L)		193		4.6 ± 5.1		2.8		0.1–34.1		
Cord plasma DHA (% phospholipids)		189		3.7 ± 1.3		3.5		1.1–7.7		
Current plasma DHA (% phospholipids)		191		2.4 ± 1.0		2.2		0.1–5.5		
Go/no-go behavioral performance										
Mean RT, correct go trials (msec)		196		479.2 ± 75.8		472.0		322.0–720.0		
Mean RT, incorrect no-go trials (msec)		196		419.5 ± 87.2		403.0		271.0–718.0		
Correct go trials (%)		196		90.1 ± 9.7		93.2		52–100		
Correct no-go trials (%)		196		64.0 ± 14.9		66.7		22–94		
**a**For women who breast-fed only.										

Associations among the concentrations of contaminants measured at birth and at time of testing are presented in Table 2. Pb, PCB-153, and Hg are moderately associated with each other, presumably because these substances are found at relatively high concentrations in traditional Inuit food and families vary in the degree to which they consume traditional food.

**Table 2:** Intercorrelations among contaminants (Pearson correlation coefficients, *r*).

Pb			PCB-153			Hg		
Chemical	Sample	Cord	Current	Cord	Current	Cord	Current
Pb		Cord		1.00		0.17**		0.28*		0.29*		0.32*		0.18**
		Current				1.00		0.04		0.25*		0.12		0.23*
PCB-153		Cord						1.00		0.45*		0.42*		0.25*
		Current								1.00		0.41*		0.55*
Hg		Cord										1.00		0.46*
		Current												1.00
*p < 0.01. **p < 0.05.														

*Behavioral performance.* Values for mean RT and rates of correct responses on the go and no-go trials are presented in Table 1. Repeated-measures analysis of variance (ANOVA) showed that, as expected, participants are more accurate on go than on no-go trials [*F*_(1,195)_ = 655.4, *p* < 0.001]. Faster mean RT for go trials is associated with more accurate detection of go (*r* = –0.31, *p* < 0.001) and no-go (*r* = –0.19, *p* < 0.001) trials. Rates of correct responses for go trials are positively associated with rates of correct responses for no-go trials (*r* = 0.39, *p* < 0.001).

**Figure 1:**
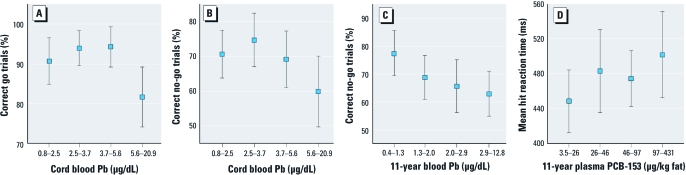
Mean ± SD behavioral performance in the go/no-go task according to quartiles of exposure: (*A,B*) cord blood Pb, (*C*) 11-year blood Pb, and (*D*) 11-year plasma PCB‑153. Outcome measures are adjusted for the potential confounders listed in Table 3.

Results from the regression analyses testing for associations between contaminant concentrations and behavioral go/no-go performance are presented in Table 3. After control for confounding factors, higher Pb concentrations in cord blood are associated with fewer correct responses for both go and no-go trials. Higher blood Pb concentrations at time of testing are associated with an increased number of false alarms in no-go trials, suggesting difficulty in inhibiting a prepotent response. This association falls just short of statistical significance when participants with blood Pb concentrations ≥ 5 μg/dL are excluded (*n* = 175; *r* = –0.18, *p* = 0.02; standardized β = –0.14, *p* = 0.078). Current plasma PCB-153 concentrations are associated with slower responses to go trials. PCB-153 concentrations in cord plasma and Hg concentrations in cord and child blood samples were not associated with any of the behavioral measures after statistical control for confounding variables (all *p*-values > 0.10). Figure 1 illustrates mean performance per quartile of exposure on each outcome significantly associated with a contaminant. Postnatal Pb and PCB-153 effects seem linear, whereas the adverse effects of prenatal Pb are mainly observed in the highest-exposed children.

**Table 3:** Associations between contaminants and behavioral performance in the go/no-go task (*n* = 196).

Pb					PCB-153					Hg				
Cord blood		Current blood		Cord plasma		Current plasma		Cord blood		Current blood	
Behavioral outcome	*r*	β	*r*	β	*r*	β	*r*	β	*r*	β	*r*	β
Mean RT, correct go trials		0.06	–0.05*a*^,b,c,d,e,f^		–0.01	0.03*a*^,b,c,d,f^		0.15**	0.04*b*^,c,e,f^		0.17**	0.18**b^,c,e,f^		0.13^#^	0.04*a*^,b,c,d,f^		0.15**	0.10*a*^,b,c,d,f^
Mean RT, incorrect no-go trials		0.14^#^	0.10*a*^,b,d,g,h^		0.01	0.03*a*^,b,d,g^		0.16**	0.07*b*^,g,h,i^		0.17**	0.06*b*^,g,i^		0.10	0.03*a*^,b,d,h,i,j^		0.20*	0.11*a*^,b,d,h^
Percent correct go		–0.12^#^	–0.21**h^,k,l^		–0.11	–0.12*k*^,l^		–0.01	0.03*h*^,i,k,l^		–0.06	–0.01*a*^,h,i,k,l^		–0.03	0.06*a*^,h,k,i,l^		–0.11	–0.02*a*^,h,i,l^
Percent correct no-go		–0.16**	–0.17**b^,d^		–0.20*	–0.16**b^,m^		0.05	0.06*b*^,i,k,m,n,o^		–0.12	–0.06*b*^,i,k,n,o^		–0.06	0.07*b*^,d,i,m,n^		–0.07	–0.03*b*^,i,k,m,o^
Values are Pearson correlation (*r*) and standardized regression (β) coefficients from multiple regression analyses. Superscripts (*a–o*) indicate control variables included in the final model for each of the regression analyses. **a**Child age. **b**Child sex. **c**Adoption status. **d**Child PCB-153. **e**Cord Hg. **f**Child DHA. **g**Child Hg. **h**Cord DHA. **i**Cord Pb. **j**Cord PCB-153. **k**Time at assessment. **l**Maternal marijuana during pregnancy. **m**Breast-feeding duration. **n**Transported from a remote village. **o**Child Pb. *p < 0.01. **p < 0.05. ^#^*p* < 0.10.																		

Testing for interactions revealed interaction effects for cord Pb with both cord PCB and cord Hg for three behavioral measures: incorrect no-go RT (standardized β: Pb × PCB = 0.14, *p* = 0.05; Pb × Hg = 0.12, *p* = 0.10 percent correct go (standardized β: Pb × PCB = –0.17, *p* = 0.04; Pb × Hg = –0.15, *p* = 0.07), and percent correct no-go trials (standardized β: Pb × PCB = –0.11, *p* = 0.12; Pb × Hg = –0.18, *p* = 0.01). Although not all these interactions reached conventional levels of statistical significance, the stratification analyses showed that the effects of cord Pb were seen primarily in the children with higher prenatal PCB and/or Hg exposures, indicating that the effects of prenatal Pb exposure were intensified by heavier PCB and Hg exposures.

*Stimulus-locked ERPs.* In repeated-measures ANOVAs with condition (go vs. no-go) as a within-subject variable, P3 amplitude at parietal electrode (Pz) was significantly larger for no-go than for go trials [10.1 vs. 5.0 μV; *F*_(1,192)_ = 119.7, *p* < 0.001], reflecting the additional resources needed to inhibit a prepotent response. No condition effect was found for N2 [frontal electrode (Fz)] latency (go: 375.9 msec; no-go: 370.7 msec) and amplitude (go: –7.4 μV; no-go: –7.8 μV; *p*-values > 0.20). Slower N2 latency was associated with slower mean hit RT (go N2: *r* = 0.35, *p* < 0.001; no-go N2: *r* = 0.30, *p* < 0.001) and with lower percent correct go trials (go N2: *r* = –0.23, *p* = 0.001; no-go N2: *r* = –0.30, *p* < 0.001). Greater P3 amplitude was significantly associated with greater percent correct go trials (go P3: *r* = 0.21, *p* = 0.003; no-go P3: *r* = 0.29, *p* < 0.001), and P3 amplitude to go trials tended to be related to greater percent correct no-go trials (*r* = 0.14, *p* = 0.051).

**Figure 2:**
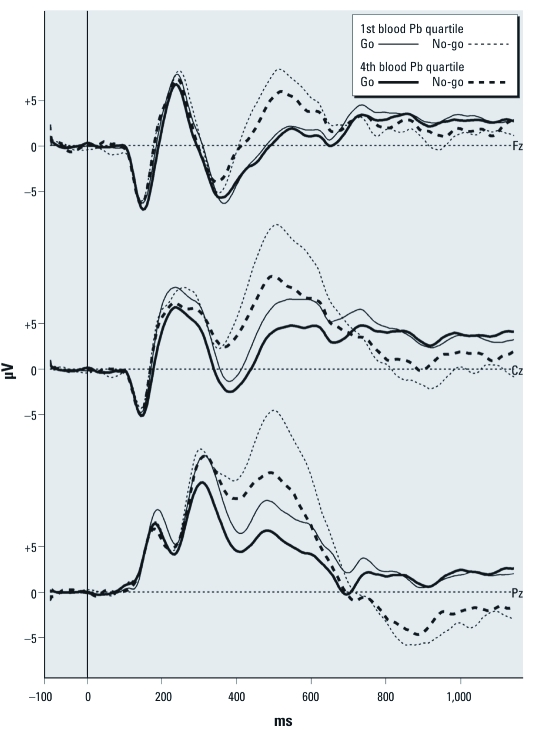
Grand average for stimulus-locked go/no-go ERPs at midline electrodes comparing participants from the first quartile of blood Pb concentrations at time of testing (range, 0.4–1.3 μg/dL; *n* = 47; thin lines) and those from the fourth quartile (range, 2.9–12.8 μg/dL; *n* = 48; thick lines) for go (solid lines) and no-go (dashed lines) trials. The N2 is the second negative component (~ 375 msec), which is more pronounced at the frontal lead (Fz). The P3 peaks around 500 msec and is larger at the parietal lead (Pz). Children with higher blood Pb concentrations show reduced activity during the P3 time window compared with children with lower Pb levels.

Results from the regression analyses testing for associations between contaminants and the stimulus-locked ERP parameters are presented in Table 4. Higher current blood Pb concentrations are associated with reduced P3 amplitude to go and no-go trials. When participants with current blood Pb concentrations ≥ 5 μg/dL are excluded, this effect is still significant for no-go P3 amplitude (*r* = –0.24, *p* = 0.001; standardized β = –0.21, *p* = 0.007) but falls short of significance for go P3 amplitude (*r* = –0.16, *p* = 0.04; standardized β = –0.09, *p* = 0.22). The effect of current blood Pb concentrations on P3 amplitude is clearly observable when contrasting the ERP averages of the bottom and top quartiles of the distributions for current blood Pb concentration (adjusted mean ± SD no-go P3 amplitude, 12.8 ± 7.0 μV vs. 7.8 ± 8.1 μV, Cohen’s *d* = –0.66; see Figure 2). The association between cord PCB-153 and go N2 latency falls short of statistical significance after controlling for maternal age at delivery (*p* = 0.053), suggesting delayed N2 latency with increasing prenatal exposure. Analyses with Hg revealed an association between current blood Hg concentrations and reduced P3 amplitude in the go condition that fell short of statistical significance after controlling for confounders (*p* = 0.052). Cord blood Hg and current plasma PCB-153 concentrations levels were not significantly associated with any of the stimulus-locked ERPs.

**Table 4:** Associations between contaminants and stimulus-locked ERP components recorded during the go/no-go task (*n* = 193).

Pb							PCB-153							Hg						
Cord blood			Current blood			Cord plasma			Current plasma			Cord blood			Current blood		
ERP parameter	*r*	β	*r*	β	*r*	β	*r*	β	*r*	β	*r*	β
N2 (Fz)																								
Go latency		–0.01		0.08*a*^,b^		0.05		0.02*b*^,c^		0.09		0.15^#a^		0.05		0.08*a*^,c^		0.08		0.05*a*^,b^		0.01		0.02*a*^,c^
No-go latency		–0.03		–0.01*d*^,e,f,g,h^		0.01		–0.01*d*^,f,g,h^		0.01		–0.11*d*^,f,g,h^		0.14^#^		0.01*e*^,f,h^		0.09		0.04*d*^,e,f,g,i^		0.16**		0.11*d*^,f^
Go amplitude		0.03		0.08*j*^,k^		0.02		0.02*d*^,j,k^		–0.03		0.00*d*^,j,k^		0.01		0.07*d*^,j^		0.09		0.13^#d,j^		0.01		0.06*d*^,j,k^
No-go amplitude		–0.00		0.02*a*^,f,j,k,l,m^		0.00		0.05*j*^,k,l,m^		–0.05		–0.01*a*^,f,j,l,m^		0.04		0.01*a*^,f,j,k,l,m^		0.02		0.03*a*^,f,j,k,l,m^		0.04		0.02*a*^,f,j,k,l,m^
P3 (Pz)																								
Go amplitude		–0.06		–0.05*c*^,h,l^		–0.20*		–0.16**h		0.03		0.08*c*^,h,l,n,o^		–0.13^#^		–0.01*c*^,h,l,n,o^		–0.08		–0.02*i*^,l,n,o^		–0.21*		–0.15^#n,o^
No-go amplitude		–0.06		–0.06*a*^,g,l,p^		–0.26*		–0.23*h		0.04		0.12*a*^,l,o,p^		–0.13^#^		–0.01*a*^,l,o,p^		–0.14^#^		–0.10*a*^,g,l,o^		–0.18**		–0.06*a*^,g,i,l,o^
Values are Pearson correlation (*r*) and standardized regression (β) coefficients from multiple regression analyses. Negative (–) associations with N2 amplitude indicate greater (more negative) amplitude. Superscripts (*a–p*) indicate control variables included in the final model for each of the regression analyses. **a**Maternal age. **b**Cord PCB-153. **c**Time at assessment. **d**Child age. **e**Adoption status. **f**Maternal binge drinking during pregnancy. **g**Breast-feeding duration. **h**Child Hg. **i**Child PCB-153. **j**Cord DHA. **k**Maternal Raven’s score. **l**Maternal smoking during pregnancy. **m**Child sex. **n**Transported from a remote village. **o**Child Pb. **p**Cord Hg. *p < 0.01. **p < 0.05. ^#^*p* < 0.10.																								

Interaction terms revealed significant interactions between current Pb and current Hg on P3 amplitude elicited in the go condition (standardized β = 0.15, *p* = 0.04) and between current Pb and current PCB-153 on P3 amplitude elicited in both the go (standardized β = 0.17, *p* = 0.02) and the no-go (standardized β = 0.13, *p* = 0.08) conditions. In all cases, the Pb effects were stronger in children with lower coexposures to the other chemicals. There were also significant interactions between cord Hg and cord PCB-153 on no-go N2 latency (standardized β = 0.16, *p* = 0.05) and no-go P3 amplitude (standardized β = –0.20, *p* = 0.02). Stratification analyses revealed that higher cord Hg resulted in slower no-go N2 latency, and smaller no-go P3 amplitude in children with higher prenatal PCB exposure, but higher no-go N2 amplitude only in children with lower prenatal PCB exposure.

*Response-locked ERPs.* Repeated-measures ANOVAs with response type (correct go, incorrect no-go) as a within-subject variable revealed significant error-related effects on mean amplitude during the ERN [fronto-central electrode (FCz): 0.3 vs. –4.0 μV; *F*_(1,166)_ = 81.6, *p* < 0.001], Pe [Cz: 1.7 vs. 8.2 μV; *F*_(1,166)_ = 127.1, *p* < 0.001], and Pc [FCz: 4.4 vs. 6.7 μV; *F*_(1,166)_ = 13.9, *p* < 0.001] latency intervals. Smaller ERN and Pe amplitudes tended to be associated with slower mean hit RT (ERN: *r* = 0.15, *p* = 0.052; Pe: *r* = –0.14, *p* = 0.07), and amplitudes of all three error monitoring components were significantly associated with higher percent correct go trials (ERN: *r* = –0.19, *p* = 0.02; Pe: *r* = 0.25, *p* = 0.001; Pc: *r* = 0.18, *p* = 0.02) and higher percent correct no-go trials (ERN: *r* = –0.23, *p* = 0.003; Pe: *r* = 0.25, *p* = 0.001; Pc: *r* = 0.16, *p* = 0.04).

**Figure 3:**
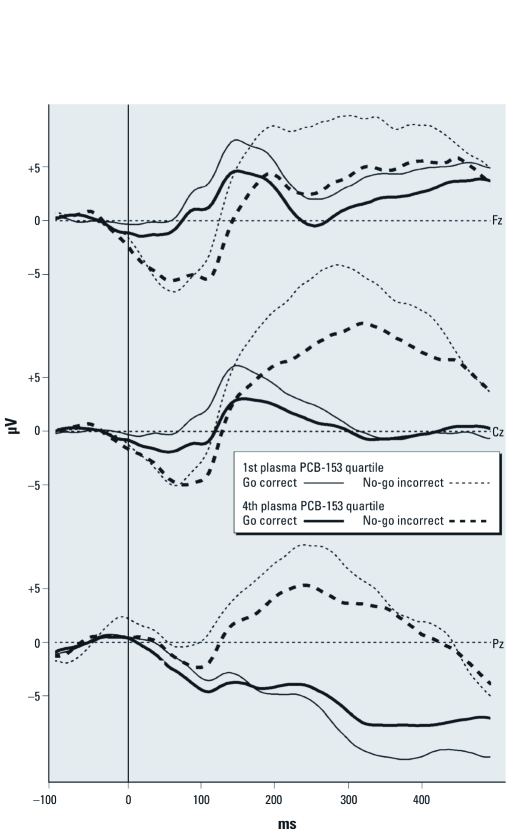
Grand average for response-locked go/no-go ERPs at midline electrodes comparing participants from the first quartile of plasma PCB-153 concentrations at time of testing (range, 3.5–26.0 μg/g fat; *n* = 46; thin lines) to those from the fourth quartile (range, 96.6–431.4 μg/g fat; *n* = 42; thick lines). Incorrect no-go trials (false alarms; dashed lines) elicit an early wave of negative voltage (ERN) followed by a large positive wave (Pe) maximal at the vertex (Cz). Correct go responses (solid lines) generate a frontocentral wave of positive voltage in the 100–300 msec (Pc) latency interval. Children with higher plasma PCB-153 concentrations show reduced activity during the Pe and Pc time windows compared with children with lower PCB-153 levels.

Results from the regression analyses testing for associations between contaminants and response-locked ERP parameters are presented in Table 5. After statistical control for confounders, higher plasma PCB-153 concentrations at time of testing are associated with reduced amplitude of the components Pe, elicited by false alarms, and Pc, elicited by correct hits. These effects are illustrated in Figure 3, which compares children from the bottom and top quartiles of current plasma PCB-153 concentrations (adjusted mean ± SD Pc amplitude, 5.5 ± 4.0 μV vs. 2.8 ± 4.8 μV, Cohen’s *d* = –0.61). Pb and Hg concentrations in cord and child blood samples were not associated with any of the response-locked ERP measures in standard regression analyses (all *p*-values > 0.10).

**Table 5:** Associations between contaminants and response-locked ERP amplitudes recorded during the go/no-go task (*n* = 166).

Pb							PCB-153							Hg						
Cord blood			Current blood			Cord plasma			Current plasma			Cord blood			Current blood		
ERP parameter	*r*	β	*r*	β	*r*	β	*r*	β	*r*	β	*r*	β
ERN (FCz)		0.03		–0.02*a*^,b,c,d^		0.09		0.11*a*^,d^		0.07		–0.03*a*^,c,d^		0.06		0.02*a*^,b,c,d^		0.12		0.11*a*^,c^		0.10		0.07*c*
Pe (Cz)		–0.11		–0.07*a*^,e^		–0.11		–0.00*a*^,e,f^		0.07		0.11*a*^,g,h^		–0.18**		–0.16**i		–0.09		0.05*a*^,e,f,g^		–0.11		–0.03*a*^,e,f^
Pc (FCz)		–0.19**		–0.12*e*		–0.10		–0.00*a*^,b,e,j^		–0.08		–0.03*a*^,b,f,g,j^		–0.23*		–0.20**g		–0.12		0.01*a*^,e,g,j^		–0.10		0.01*a*^,b,e,f,j^
Values are Pearson correlation (*r*) and standardized regression (β) coefficients from multiple regression analyses. Negative (–) associations with ERN amplitude indicate greater (more negative) amplitude. Superscripts (*a–j*) indicate control variables included in the final model for each of the regression analyses. **a**Child sex. **b**Adoption status. **c**Transported from a remote village. **d**Cord Hg. **e**Child PCB-153. **f**Breast-feeding duration. **g**Cord Pb. **h**Child Hg. **i**Child Pb. **j**Child age. *p < 0.01. **p < 0.05.																								

The effects of current plasma PCBs on response-locked ERPs did not interact with the other contaminants (all *p*-values > 0.15).

## Discussion

In this study we examined the association of developmental exposure to Pb, PCBs, and MeHg with response inhibition in 11-year-old children using ERPs recorded during a visual go/no-go task. Blood Pb concentrations at time of testing were associated with higher rates of false alarms during the task and with reduced amplitude of the P3 wave elicited by go and no-go stimuli, suggesting reduced allocation of cognitive resources for task processing. Surprisingly, the effects of current Pb on P3 amplitudes were stronger in children with lower current MeHg and PCB exposures, which suggests that coexposure to the other contaminants may make the adverse Pb effects on these end points more difficult to detect. By contrast, plasma PCB-153 concentrations at time of testing were associated with slower RTs to go trials and with reduced amplitude of the Pe/Pc components, which are believed to reflect postresponse brain activity involved in error monitoring. Higher cord blood Pb concentrations were associated with poorer performance during the task (although not with any of ERP parameters), and the behavioral effects were markedly stronger in children with higher levels of prenatal exposure to MeHg and to PCBs. Cord PCB-153 levels were marginally associated with delayed latency of the N2 component in the go but not in the no-go condition.

We previously assessed the relation of postnatal Pb exposure to ERPs recorded during a simple auditory oddball detection paradigm and found no effect of Pb on the P3 component recorded during this task at school age (Boucher et al. 2009b). The finding of decreased P3 amplitude to go and no-go stimuli as a function of current blood Pb concentrations in the present analyses suggests that adverse effects of childhood Pb exposure are detected more readily when higher-order, executive processes (i.e., response inhibition) are elicited. This inference is also supported by the finding that the association of postnatal Pb with P3 amplitude was more marked in the no-go than in the go condition. Impulse control and inhibition have been shown to be specifically impaired in Pb-exposed animals (Brockel and Cory-Slechta 1998), and evidence is accumulating that these aspects of cognition are among the most sensitive to Pb exposure in children. Higher ratings of impulsive and inattentive behavior, impaired response inhibition, and increased risk of attention deficit hyperactivity disorder have been reported in samples of children with mean blood Pb concentrations as low as ≤ 5 μg/dL (Froehlich et al. 2009; Nicolescu et al. 2010; Stewart et al. 2006; Surkan et al. 2007). These effects suggest enhanced vulnerability of the prefrontal cortex to Pb neurotoxicity during childhood, a hypothesis that has been supported by imaging studies revealing specific structural (Cecil et al. 2008) and metabolic (Cecil et al. 2011; Meng et al. 2005) Pb-associated alterations in this region of the brain. The present study adds to this evidence by reporting an inverse relation between blood Pb levels (< 5 μg/dL) and amplitude of the no-go P3 ERP component, which is believed to be generated within the prefrontal cortex (Davis et al. 2003). These effects, observed at such low Pb levels, also strengthen the arguments for revising the blood Pb concentrations considered “acceptable” by public health agencies from 10 μg/dL (Centers for Disease Control and Prevention 1991; Health Canada 1994) to a lower value. The mean blood Pb concentration in this study is similar to that reported for preschool children from the general U.S. population during the mid-1990s (Jones et al. 2009), which adds to the growing body of evidence that a large proportion of children exposed at levels considered safe under current public health recommendations actually show subtle adverse effects from Pb neurotoxicity (e.g., Chiodo et al. 2004; Lanphear et al. 2000).

In this study, cord blood Pb concentrations were associated with increased errors in both the go and no-go trials. This pattern of results may reflect an effect of prenatal Pb exposure on attention and cognitive function generally rather than the specific effect on response inhibition seen in relation to current Pb only on the no-go trials. This may explain why no effect of prenatal Pb exposure was observed on the ERP measures. Adverse effects of prenatal Pb exposure on attention have been reported in previous assessments conducted on this same cohort of children (Boucher et al. 2009b; Plusquellec et al. 2007). Although no adverse behavioral effects were seen in relation to prenatal PCB exposure on this relatively simple task, the ERP data suggest impairment in processing that would be expected to affect performance in a more challenging task.

The delayed RTs and reduced Pe/Pc amplitudes seen in relation to postnatal PCB exposure in this study suggest that PCB exposure alters the neural processes involved in error monitoring. Neural generators of the Pe have been localized, notably, in the rostral portion of the anterior cingulate cortex (ACC) (Herrmann et al. 2004; Van Veen and Carter 2002). Lesions involving the rostral ACC have been associated with impairment in the regulation of cognitive control after response conflict, leading to poor context-dependent adjustments of behavior (Di Pellegrino et al. 2007). Poorer behavioral regulation in relation to contextual information, such as previous errors, might account for the high sensitivity of delayed reinforcement paradigms and of tasks assessing planning and response inhibition to early PCB exposure (Jacobson and Jacobson 1996, 2003; Lilienthal et al. 1990; Rice 1999; Stewart et al. 2005, 2006; Vreugdenhil et al. 2004).

Most previous birth cohort studies on PCB neurotoxicity have reported adverse effects of prenatal but not postnatal PCB exposure on cognition (e.g., Boucher et al. 2009a; Jacobson and Jacobson 1996; Jacobson et al. 1985). The effects associated with postnatal PCB exposure in this study are likely explained by the typically longer period of breast-feeding in this Inuit sample and the substantial quantities of PCB-contaminated traditional Inuit food eaten by these children than that seen in U.S. and southern Canadian children. This extended breast-feeding likely has led to postnatal transmission of much larger quantities of these contaminants than in other PCB cohort studies. Adverse neurobehavioral effects of postnatal PCB have been demonstrated in experimental studies with animals (e.g., Rice 1999), supporting the plausibility of adverse effects from postnatal PCB exposure when the levels are sufficiently high.

Although cord and child Hg levels were correlated with some of the behavioral and ERP measures, virtually none of these associations remained significant once other contaminants or covariates were included in the analysis. The synergistic effects between cord Hg and cord PCB seen on some neurobehavioral end points by Grandjean et al. (2001) were not seen consistently in this study. Although the stratified analyses indicated effects of cord Hg on three ERP measures in the children with heavier prenatal PCB exposures, the effect on a fourth ERP measure was stronger in children with lower prenatal PCB exposure. It has been suggested that statistical control for seafood nutrients, such as the polyunsaturated fatty acid DHA, may provide a better estimation of the effects of MeHg exposure (Budtz-Jørgensen et al. 2007). The absence of clear effects even after statistical control for DHA in the present study therefore provides additional support for the conclusion that response inhibition and error monitoring are not specific targets of MeHg neurotoxicity in children.

## Conclusion

This study was designed to examine the neurophysiological correlates of response inhibition impairment associated with Pb and PCB exposures in children. The results suggest that childhood Pb exposure specifically impairs the allocation of the resources needed for correctly inhibiting a prepotent response, which results in increased impulsivity. By contrast, postnatal PCB exposure appears to affect the processes associated with error monitoring, an aspect of behavioral regulation required to adequately adapt to the changing demands of the environment, which has been localized within the ACC. This effect results in reduced task efficiency and may play a role in the PCB-related cognitive impairments reported in previous studies. The present data demonstrate the utility of ERPs in advancing understanding of the mechanisms underlying the neurotoxicity of environmental contaminants.
